# Maternal mid‐upper arm circumference during pregnancy and linear growth among Cambodian infants during the first months of life

**DOI:** 10.1111/mcn.12951

**Published:** 2020-08-24

**Authors:** Daniel Edem Kpewou, Etienne Poirot, Jacques Berger, Somphos Vicheth Som, Arnaud Laillou, Selamawit Negash Belayneh, Frank T. Wieringa

**Affiliations:** ^1^ Maternal, Newborn and Child Health and Nutrition Section United Nations Children's Fund (UNICEF) Phnom Penh Cambodia; ^2^ Institute of Research for Development (IRD) UMR Nutripass IRD‐UM2‐UM1 Montpellier France; ^3^ Department of Nutrition Reproductive and Child Health Alliance (RACHA) Phnom Penh Cambodia

**Keywords:** Cambodia, infant, maternal MUAC, stunting

## Abstract

Stunting prevalence among children under 5 years remains high in Cambodia, affecting about one‐third of children. In most low‐ and middle‐income countries, linear growth faltering of young children starts in the womb. The 1,000‐days window of opportunity to improve child nutritional status includes pregnancy, as maternal nutritional status is an important determinant of birthweight and child development. In Cambodia, nutritional status of pregnant women is poor, with some studies reporting >20% of pregnant women having a low mid‐upper arm circumference (MUAC < 23 cm). Few studies have investigated associations between maternal nutritional status during pregnancy and neonatal growth. Using data from a Cambodian cohort study conducted from 2016 through 2018 in selected districts of Phnom Penh, Kratie, and Ratanakiri provinces, we investigated associations between maternal MUAC during pregnancy as indicator of maternal nutritional status and their offspring linear growth during early life. Multivariate regression models were used to assess the associations between maternal MUAC during the last trimester of pregnancy and infant's length‐for‐age z‐scores during the first 3.5 months of life. Maternal MUAC was significantly associated with infant's length‐for‐age z‐scores (regression coefficient 0.06, 95% CI [0.03, 0.09]). Infants born from mothers with a low MUAC during pregnancy had a 1.6 times higher risk (odds ratio 1.621, 95% CI [0.998, 2.636]) of being stunted during the first 3.5 months of life compared with infants born from mothers with a MUAC >23 cm. This study underlines the importance of optimum maternal MUAC during pregnancy for optimal infant growth. Interventions that aim to tackle stunting in infants should integrate improving maternal MUAC during pregnancy.

**Key messages**

- Maternal MUAC during the last trimester of pregnancy is associated with infant length‐for‐age z‐scores during the first 3.5 months of life.
- Stunting starts before the birth of the child, and interventions to prevent this should be starting during antenatal care, as infants born from mothers with low MUAC (<23 cm) during pregnancy had a 1.6% higher risk for being stunted than infants born from mothers with normal MUAC.
- Cambodia should introduce screening for maternal malnutrition using mid‐upper arm circumference during antenatal care (ANC) and target mothers with MUAC below 23 cm with specific nutrition counselling and support.

## 1 INTRODUCTION

Pregnancy puts women at a higher risk of malnutrition due to higher nutrient requirements and leads to deficiencies in both micronutrients and macronutrients, if dietary intake is not increased accordingly (Nguyen, 2019). Malnutrition during pregnancy is not only detrimental for maternal health but also affects foetal and neonatal health (Williamson, 2006). Maternal malnutrition can be apparent in anthropometric indicators such as a low mid‐upper arm circumference (MUAC) or in biochemical tests, revealing, for example, anaemia or a specific micronutrient deficiency. A recent study conducted in India reported increased risks of low birthweight for offspring in anaemic pregnant women, an effect that was enforced if women were also underweight (Patel et al., 2018). Hence, maternal nutritional status before and during pregnancy is vital for a healthy pregnancy outcome (Kramer & Victora, 2001).

Most studies that considered maternal nutritional status and pregnancy outcomes report on birthweight rather than looking at later development of the infant, such as linear growth (Papathakis, Singh, & Manary, 2016). Even though it was proven that birthweight is a strong predictor for infant growth, there is not enough evidence to suggest that nutritional status of a mother during pregnancy has a long‐term impact on the child's growth (Queiroz, Assis, Pinheiro, & Ribeiro, 2012). Linear growth faltering, which leads to stunting, is an indicator of chronic malnutrition. Linear growth faltering starts often during the first months of infancy and settles during the first 2 years of life. According to the World Health Organization (WHO) 2006 Child Growth Standards, if a child falls below −2 standard deviation (SD) on the recommended length/height‐for‐age growth charts, s/he is considered stunted (WHO, 2006). Stunting is linked to many causal factors such as maternal nutritional status, dietary intake, infections, socioeconomic status, micronutrient deficiencies, and the environment (Dewey, 2016). Impaired cognitive development, increased vulnerability to chronic diseases in adulthood, lower attained schooling, and reduced adult income due to reduced capacity in completing developmental potential have all been associated with stunting (Black et al., 2008; Ikeda, Irie, & Shibuya, 2013; Victora et al., 2008). As maternal nutritional status affects foetal growth and birthweight (Victora et al., 2008), it is important to understand how this relates with the development of linear growth retardation in infancy and early childhood.

Although no clear cut‐offs have been established, MUAC has been increasingly used to assess the nutritional status of adults, especially pregnant women, as it offers the merits of being a simple measure that can be performed in both facility‐ and community‐based settings, requiring minimal equipment and training, compared with body mass index (Kerac, McGrath, & Seal, 2010; Tang et al., 2016). Moreover, MUAC was shown to be a good predictor for low birthweight (Ververs, Antierens, Sackl, Staderini, & Captier, 2013). Yet the relationship between maternal MUAC during pregnancy and the risk of infant stunting is not fully understood and remains understudied.

Notwithstanding the significant gains that Cambodia has made over the years regarding some health and development indicators, such as the reduction in infant and under‐five mortality and increased access to sanitation (indicated by increased number of households with toilet facilities and a place for hand washing; National Institute of Statistics, 2015), malnutrition remains a major public health concern. In 2014, 10% and 32% of Cambodian children under 5 years were wasted and stunted, respectively (National Institute of Statistics, 2015). In addition, Som et al. (2018) reported that >20% of pregnant women had a MUAC < 23 cm, reflecting malnutrition. Persistent undernutrition is one factor that may offsets the recent achievements that the nation made with regards to health and development and thus, threatens the realization of the 2030 Sustainable Development Goals.

Cambodia has shown commitments towards safeguarding the health of pregnant women through various policies that ensure access to public health programmes and universal health coverage as well as interventions that focus on access to quality healthcare services and improving healthcare financing. For example, the National Nutrition Programme of Cambodia provides basically 90 iron and folic acid supplements combined with nutritional counselling for all pregnant women in health facilities. Apart from iron–folate supplements, antenatal visits are also a point of access to other services such as provision of deworming pills, tetanus vaccination, health checks, counselling for high risk activities such as smoking and drug consumption, and information on hygiene, maternal, and newborn feeding practices.

To understand the impacts of these interventions on infant health and to design new interventions, it is vital to explore associations between maternal nutritional status and the risk of infant stunting. This study therefore seeks to assess the relationship between maternal MUAC during pregnancy and linear growth faltering (stunting) in infants aged 0 to 3.5 months in selected districts of the Phnom Penh and north‐east regions of Cambodia.

## 2 METHODS

### 2.1 Data sources

The present study used data collected under the Cambodian longitudinal study, “MyHealth,” to quantify the impact of maternal MUAC on linear growth of infants during the first 3.5 months of life. MyHealth is a project originated and funded by the Cambodian Ministry of Health, UNICEF, and the French Institute of Research for Development. The project obtained ethical approval from the National Ethical Committee for Health Research under the full name of “The Cambodian Health and Nutrition Monitoring Study.” The prime objective was to collect in‐depth data on women and children under 5 years of age in Cambodia to evaluate national health and nutrition interventions. This ongoing project collected data in six districts within three provinces in the country: one district in Phnom Penh province (Russei Kaev district), two districts in Kratie province (Chitr Borie and Krong Kratie districts), and three districts in Ratanakiri province (Ou Chum, Krong Ban Lung, and Bar Kaev districts). Kratie and Ratanakiri provinces are part of the north‐east region of Cambodia. A minimum sample size of 1,200 children per province was set, with an expected reduction in the prevalence of stunting of 6% (from 32% to 26%) during a 24‐month period of follow‐up with a precision of 3% and an expected dropout rate of 20%. Informed consent was obtained from all participants with consent for child participation obtained through the adult primary caregiver (usually the mother). The recruitment process was implemented with a help from village health support groups who assisted in identifying prospective participants and in collaboration with community midwives and prepared lists of prospective eligible women and children who were later invited to join the study according to the order on the list until the minimum sample size was reached. The pregnant women were recruited into the study at any stage of their pregnancy. The overall cohort population of the MyHealth project is composed of three distinct groups: (a) married nonpregnant women at reproductive age (between 14 and 40 years old), (b) pregnant women, and (c) children below 2 years of age at baseline data collection. Women and children (aged between 0 and 24 months at baseline) were revisited every 3 months during the first year (2016), every 4 months during the second year (2017), and once per year thereafter. During each follow‐up, new pregnant women and infants of old pregnant women from the previous follow‐up were invited to participate in the study. Data were collected using a tablet‐based questionnaire. The data collection includes anthropometric measures of both children and women, mothers'/caretakers' health knowledge and practices, households' environment, and child's cognitive development.

Trained field staff conducted anthropometric measurements using regularly calibrated equipment. The weight of the children was measured using SECA mother–infant digital scales (SECA UK.), being calibrated after each fifth measure with a precision point of 100 grams. Length/height boards with standing plates and moveable head boards from UNICEF supply division were used to measure the length of children to the closest 1 mm. MUAC of pregnant women was measured using plastic simple MUAC tape incapable of stretching and unresponsive to high temperatures (UNICEF Supply Division, Copenhagen, Denmark). All measurements were taken in duplicates and averages computed to ensure accuracy. Other information collected from pregnant women included household and demographic characteristics, water, sanitation and hygiene (WASH) practices, attendance of antennal clinics, dietary diversity in the past 24 hr, and health knowledge. Additional information collected during the study included infant and young child feeding practices, access to national health campaigns, and cognitive development of the newborn.

Infant and young child feeding practices were assessed per UNICEF and WHO indicators for appropriate breastfeeding (WHO, 2008), including early initiation of breastfeeding, breastfeeding status, duration of exclusive breastfeeding, and 24‐hr recall on breastfeeding and bottle frequency. The methodology of MyHealth longitudinal study presented in more details in our previous publications (Hondru et al., 2019; Manzoni et al., 2019; Som et al., 2018; Wieringa et al., 2018).

In the present study, we used a subsample of the “MyHealth Study,” by selecting all pregnant women at third trimester of pregnancy and the infants that resulted from these pregnancies. Data were collected between March 2016 and September 2018. In this analysis, the Kratie and Ratanakiri provinces were combined to form the North‐East region. As such, two regions, the North‐East and Phnom Penh regions, were used in the analysis. The inclusion criteria for the present study included (a) available pregnant women MUAC measurements (taken during the third trimester of pregnancy), (b) availability of anthropometric measures (weight and length) for their infants (available at the closest follow‐up round after delivery), (c) infants who were singleton, and (d) infants who were not suffering from any medical complications or deformities. Gender, weight, and length were recorded for all children.

### 2.2 Data processing

The infant's age in months was calculated by subtracting the date of the follow‐up visit from the date of birth of the infant. We calculated length‐for‐age z‐scores (LAZ) using WHO 2006 standards for children 0–59 months (WHO, 2006) using WHO Anthro software (version 3.2.2, January 2011).

With the aim to describe the effect of mother's nutritional status during pregnancy on early child growth, we used infants’ length‐for‐age z‐scores which were computed using infants’ length measurements closest to infants’ birth dates, as the outcome. Since this study was restricted to the first follow‐up after birth, the age of the infants during assessment ranged from a few days to 3.5 months. We have assumed that the first length measure will more likely reflect the effect of maternal MUAC during pregnancy than later length measurements that probably would have been affected by other environmental factors.

**Figure 1:**
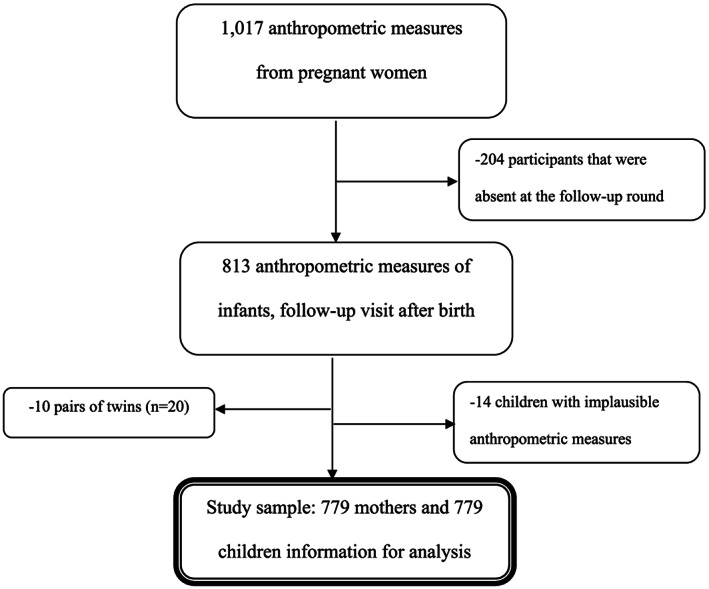
Participant recruitment and enrolment flowchart

If a pregnant woman had more MUAC measurements available in the third trimester, the last MUAC measurement before delivery was taken for the current analysis. Overall, MUAC data were available from 1,017 pregnant women, regardless of gestational age, from which 813 of these pregnant women returned with their infant at the first follow‐up after birth. A total of 779 women and their infants were included in the final statistical analysis of the current study (Figure 1).

In Cambodia, antenatal care visits are part of the compulsory services for pregnant women. As part of our study, data collected on antenatal care attendance during pregnancy was considered as a binary variable whether the pregnant women had ever attended antenatal care visits or not.

Taking into consideration the age of the children included in this study (<6 months of age), exclusive breastfeeding should be the only method used for feeding the infant, and this was considered in the analysis as a binary variable denoted whether or not the infant was being exclusively breastfed or not.

As described by Food and Agriculture Organization and FHI 360 (2016), women's dietary diversity was computed through the women's dietary diversity score with nine food groups: (a) starchy staples, (b) legumes and nuts, (c) dairy, (d) organ meat, (e) eggs, (f) flesh meat and fish, (g) dark green leafy vegetables, (h) other vitamin A‐rich vegetables and fruits, and (i) other fruits and vegetables. The score is the sum of the nine categories of food groups consumed in the past 24 hr. The score represents the sum of these categories, with lower values indicating nutritionally inadequate dietary diversity. Household wealth index, a composite measure of a household's living standard, was constructed using principal component analysis, as described by Filmer and Prichett (2001). The index includes materials used for housing construction, type of cooking fuel, and ownership of selected assets such as radio, television, refrigerator, and sanitation (O'Donnell, Van Doorslaer, Wagstaff, & Lindelow, 2008). Once the index was created, households were divided into quintiles (poorest, poor, middle, richer, richest). Mother's education was divided in three groups: no education if the mother had not attended school (none), informal or primary school only (primary), and higher education if otherwise (secondary and above).

We acknowledge that other covariates can influence the outcome being measured. However, the available data did not include information on these, and thus, we considered this as a limitation to the present study. Birthweight was not systematically recorded by the MyHealth project but by the health system. Morbidity data were not available for all infants in the first 3 months especially in the rural areas. Child immunization was collected at most follow‐ups in the cohort study, but coverage for BCG, given at birth, is almost 100% and therefore not meaningful for statistical analysis.

### 2.3 Statistical analyses

Stunting was defined as z‐scores for length‐for‐age below −2 SDs, whereas a suboptimal nutritional status for mothers was defined as MUAC measures below 23 cm (Ververs et al., 2013). The Shapiro–Wilk test was used to determine the normality of pregnant women MUAC and infant LAZ data. Through bivariate analysis, the relationship between maternal MUAC and other variables (women's diet diversity score, infant's gender [Male/Female], infant's age, exclusive breastfeeding status [Yes/No], region, maternal educational level, household wealth index, antenatal care attendance, and receiving nutrition and breastfeeding information for the infant during antenatal visit) and infant's LAZ at ages 0 to 3.5 months was assessed (Table 2). Variables with a significant effect (*p* < .1) in the bivariate analysis were further included in the multivariate linear regression models. Through this multivariate linear regression analysis, it was possible to estimate the potential effect of covariables on the early linear growth. In the multivariable linear regression, maternal MUAC measured during pregnancy was included in the model as a continuous variable and was the main predictor of the infant's LAZ at 0 to 3.5 months of age. Covariates included were women's diet diversity score, infant's gender (Male/Female), infant's age, region, maternal educational level, and household wealth index. Four models (table 3) were constructed to assess whether the impact of maternal MUAC during pregnancy on infant LAZ was sustained when other covariates were introduced. In the first model, a bivariate analysis was performed with mother's MUAC during pregnancy as the predictor of infant's LAZ at 0 to 3.5 months. Model 2 introduced mother's dietary diversity at pregnancy, infant's gender, age, and region. Model 3 added mother's education to the second model, whereas in Model 4, household wealth index was added. Variables were sequentially entered into the models in groups to analyse the effect on the infant's nutritional status, while maintaining the main exposure: mother's MUAC. Mother's MUAC during pregnancy, mother's dietary diversity, and infant's age in months were considered as continuous in all models. Household wealth index, using the quintiles (1st = poorest, 2nd = poorer, 3rd = middle, 4th = richer, and 5th = richest households), infant's gender, mother's education, and region were considered as categorical variables. These categorical variables were entered into the models as dummy variables. The reference groups were male gender, Phnom Penh as compared with north‐east region, no education as compared with the other types, and poorest wealth index compared with other wealth levels. Breastfeeding status, receiving antenatal care during pregnancy, receiving nutrition, and breastfeeding information during antenatal care were insignificant variables in the bivariate analysis and therefore were not included in the multivariate models. Furthermore, adjusted logistic regression models were used to assess the risk of becoming stunted in the period of 0 to 3.5 months in which maternal MUAC was included as a categorical variable. We ensured the absence of multicollinearity among our independent variables by computing the variance inflation factor for all these variables and also by examining the coefficients and standard errors in the regression models. Missing information on mother's education (4% missing) and household wealth index (5% missing) were resolved through multiple imputations using chained equations for nominal variables in regression analysis. The missing values were replaced by the fitted estimates of the computed method for multiple imputation (Lee & Carlin, 2010; Rubin, 1987; Van Buuren, 2007).

A two‐sided significance level (*p* value ≤ .05) was used. Results are presented as mean ± SD and β coefficient for continuous outcomes and percentages and odds ratios for categorical outcomes. All statistical analyses were performed using STATA software version 13.0 (Stata Corp, College Station, TX, USA).

## 3 RESULTS

A total of 779 mothers and their 779 children were included in this analysis. The mean maternal MUAC was 25.1 ± 2.71 cm (Mean ± SD). Overall, 22.7% of all pregnant women were found to have a MUAC <23.0 cm. The mean age of children was 1.75 ± 0.95 months. There was no significant difference between the number of boys and girls in the sample (Males = 53.8% vs. females = 46.2%; Data S1). Mean LAZ of the infants was −0.71 ± 1.17 SDs, with 11.8% of infants being stunted (Table 1).

**Table 1:** General characteristics of children and pregnant women in the study

Characteristics	*n* (%)
Characteristics of infant (*n* = 779)	
Infant's age (months)	Mean (1.75), +SD (+0.96)
Infant's gender	
Male	419 (53.8)
Female	360 (46.2)
Length‐for‐age	Mean (−0.71), SD (+1.17)
Stunting	
Stunted	92 (11.81)
Not stunted	687 (88.19)
Infant being exclusively breastfed	
Yes	727 (93.3)
No	52 (6.7)
Characteristics of pregnant woman (*n* = 779)	
Pregnant woman's MUAC (cm)	Mean (25.11), SD (+2.71)
Antenatal attendance	
Yes	682 (87.6)
No	97 (12.4)
Received nutrition information at pregnancy	
Yes	419 (53.8)
No	360 (46.2)
Received breastfeeding information at pregnancy	
Yes	515 (66.1)
No	264 (33.9)
Pregnant women's education level*	
None	172 (23.0)
Primary school only (1 to 6 years)	294 (39.3)
Secondary school & above (7 years+)	282 (37.7)
Household wealth index	
Poorest (1st quintile)	172 (23.2)
Poorer (2nd quintile)	165 (22.3)
Middle (3rd quintile)	154 (20.8)
Richer (4th quintile)	151 (20.4)
Richest (5th quintile)	98 (13.2)
Mother's dietary diversity score at pregnancy	
1 food group	1 (0.1)
2 food groups	29 (3.7)
3 food groups	164 (21.1)
4 food groups	213 (27.3)
5 food groups	157 (20.2)
6 food groups	112 (14.4)
7 food groups	54 (6.9)
8 food groups	25 (3.2)
9 food groups	24 (3.1)

Note.

* Have missing values.

There was a positive, linear association between mother's MUAC during pregnancy and infant's LAZ at ages 0 to 3.5 months (*p* < .001; Table 2). Infants born from mothers with a low MUAC had a significant higher stunting prevalence (16.4%) than infants born from mothers with a MUAC >23 cm (10.5%, *p* = .032). Moreover, mother's dietary diversity score during pregnancy and mothers' education were positively associated with the infant's LAZ (*p* < .05 for both), whereas infant's age was negatively associated with LAZ.

**Table 2:** Bivariate analysis infants' (aged 0 to 3.5 months) LAZ and independent variables using imputed data

Variable	Coef.	95% CI	*p* value
Mother's MUAC at pregnancy (cm)	0.067	[0.037, 0.097]	.000
			
Mother's DDS at pregnancy*	0.089	[0.039, 0.141]	.001
			
Household wealth index			
Poorest (1st quintile)	*Ref.*		
Poorer (2nd quintile)	−0.099	[−0.350, 0.152]	.438
Middle (3rd quintile)	0.091	[−0.162, 0.344]	.478
Richer (4th quintile)	0.149	[−0.107, 0.405]	.254
Richest (5th quintile)	0.059	[−0.236, 0.356]	.693
			
Pregnant woman's education level			
None	*Ref.*		
Primary school only (1 to 6 years)	0.192	[−0.026, 0.411]	.085
Secondary school & above (7 years+)	0.402	[0.181, 0.623]	.000
			
Age of infant in months	−0.107	[−0.194, 0.022]	.014
Gender of infant			
Male	0.004	[‐0.161, 0.169]	.962
Female	*Ref*		
			
ANC attendance			
Yes	*Ref*		
No	−0.165	[−0.415, 0.084]	.194
			
Received nutrition info at pregnancy			
Yes	*Ref*		
No	0.068	[−0.097, 0.234]	.415
			
Received breastfeeding inform at pregnancy			
Yes	*Ref*		
No	−0.005	[−0.179, 0.169]	.955
			
Region			
Phnom Penh	*Ref*		
North‐east	−0.239	[−0.435, 0.044]	.016
			
Infant exclusively breastfed			
Yes	*Ref*		
No	0.120	[−0.147, 0.387]	.378

Note.

* considered as a continuous variable in this analysis. *p value i*s statistically significant at *p* < .05.

Abbreviations: CI, confidence interval; Coef., coefficient; DDS, dietary diversity score; LAZ, length‐for‐age z‐scores; MUAC, mid‐upper arm circumference; Ref.; reference category.

In all the linear regression models, mother's MUAC during pregnancy was significantly associated with infant's LAZ (*p* < .001 for all), regardless of addition of other covariates into the models. Overall, a 0.1‐cm increase in mother's MUAC during pregnancy was associated with an increase of 0.06 z‐scores in the infant's LAZ. Both maternal dietary diversity score during pregnancy and infant's age were significantly associated with the infant's LAZ (*p* < .05 for both) in all models. In the Model 2 of the multivariable linear regression (Table 3), increasing dietary diversity by one food item during pregnancy was associated with an 0.08 increase in infant's LAZ. In contrast, infant's age was negatively associated with LAZ with each additional month in age being associated with a 0.12 SDs decrease in LAZ (*p* < .01).

**Table 3:** Multivariable linear regression showing the relationship between maternal MUAC measured during the third trimester of pregnancy and the length‐for‐age z‐score of infants aged from 0 to 3.5 using imputed data

	Model 1		Model 2		Model 3		Model 4	
	Coef. (95% CI)	*p*	Coef. (95% CI)	*p*	Coef. (95% CI)	*p*	Coef. (95% CI*)*	*p*
Mother's MUAC at pregnancy (cm)	0.067 [0.037, 0.097] *R* ^2^ = .024	.000	0.063 [0.033, 0.093]	.000	0.061 [0.031, 0.091]	.000	0.061 [0.030, 0.091]	.000
								
Mother's dietary diversity			0.077 [0.026, 0.128]	.003	0.063 [0.012, 0.115]	.016	0.068 [0.016, 0.120]	.010
Gender of infant Male Female		*Ref*	−0.023 [−0.185, 0.140]	.784	−0.031 [−0.193 0.131]	.710	−0.035 [−0.198, 0.126]	.688
Infant's age (months)			−0.118 [−0.202, 0.033]	.006	−0.121 [−0.205, 0.036]	.005	−0.122 [−0.207, 0.037]	.005
Region Phonm Penh North‐east			−0.170 [−0.364, 0.024]	*Ref* 0.086	−0.140 [−0.337 0.057]	*Ref* 0.162	−0.159 [−0.364, 0.046]	*Ref* 0.130
			*R* ^2^ = .049					
Mother's education None Primary school only (1 to 6 years) Secondary school & above (7 years+)					0.145 [−0.071, 0.362] 0.309 [0.087, 0.531]	*Ref* 0.811 0.006	0.150 [−0.068, 0.369] 0.170 [0.057, 0.283]	*Ref* 0.178 0.006
					*R* ^2^ = .060			
HH. wealth index Poorest (1st Quintile) Poorer (2nd Quintile) Middle (3rd Quintile) Richer (4th Quintile) Richest (5th Quintile)							−0.165 [−0.412, 0.082] −0.045 [−0.303, 0.212] −0.060 [−0.323, 0.203] −0.197 [−0.509, 0.114]	*Ref* 0.191 0.732 0.656 0.214
							*R* ^2^ = .062	

*Note.* *p value* is statistically significant at *p* < .05. Covariates in Models 2–4: mother's MUAC during pregnancy, mother's dietary diversity and infant's age in months were considered as continuous in all models all other variables were considered as categorical. Food groups for WDDS: starchy staples, legumes and nuts, dairy, organ meat, eggs, flesh meat and fish, dark green leafy vegetables, other vitamin A‐rich vegetables and fruits, and other fruits and vegetables.

Abbreviations: CI, confidence interval; Coef., coefficient; HH, household; MUAC, mid‐upper arm circumference; Ref, reference; WDDS, women's dietary diversity score.

Finally, education of the mother up to secondary school level and above (Table 3) was significantly associated with infant LAZ (*p* < .01). Holding all covariates constant, the expected LAZ of an infant whose mother had secondary school and above was 0.17 more than the expected LAZ of an infant whose mother has no formal education.

The association between household wealth index and infant's LAZ was however not statistically significant.

Infants from mothers with a low MUAC during pregnancy had a 1.6 times (odds ratio 1.621, 95% CI [0.998, 2.636]) higher risk for being stunted during the first months of life, although this risk was borderline significant in the adjusted model (Table 4, *p* = .051). Also, infants from mothers with secondary school or higher level of education had half the risk of being stunted compared with infants born from mothers with no formal education.

**Table 4:** Comparing mother's MUAC at pregnancy and other factors with infant stunting using imputed data

Factor	Category	COR	95% CI	*p value*	AOR	95% CI	*p value*
	MUAC <23.0 cm	1.676	[1.002, 2.752]	.032	1.621	[0.998, 2.636]	.051
Mother's MUAC at pregnancy	MUAC >23.0 cm	*Ref.*					
Pregnant woman's education level	No formal education	*Ref.*					
	Primary school (1–6 years)	0.810	[0.456, 1.451]	.441	0.737	[0.425, 1.281]	.280
	Secondary school & above (7 years+)	0.498	[0.262, 0.946]	.020	0.467	[0.249, 0.875]	.017
Household wealth index	Poorest (1st Quintile)	0.624	[0.309, 1.243]	.149	0.580	[0.303, 1.112]	.101
	Poorer (2nd Quintile)	*Ref.*					
	Middle (3rd Quintile)	0.752	[0.375, 1.488]	.381	0.869	[0.449, 1.682]	.677
	Richer (4th Quintile)	0.589	[0.278, 1.216]	.124	0.711	[0.356, 1.422]	.335
	Richest (5th Quintile)	0.411	[0.145, 1.028]	.041	0.466	[0.189, 1.153]	.099
Infant's gender	Male	*Ref.*					
	Female	1.448	[0.935, 2.240]	.097	1.573	[1.008, 2.454]	.046
Infant's age (months)	‐	0.959	[0.762, 1.207]	.720	0.964	[0.758, 1.224]	.762

*Note.* *p value* is statistically significant at *p* < .05. Continuous variables: infant's age in months; Categorical variables: mother's MUAC at pregnancy, mother's education at pregnancy, household wealth index, infant's gender.

Abbreviations: AOR, adjusted odds ratio; CI, confidence interval; COR, Crude odds ratio; MUAC, mid‐upper arm circumference

## 4 DISCUSSION

The present study shows that there is a consistent association between maternal MUAC during pregnancy and linear growth and stunting prevalence of Cambodian infants during the first months of life. Although maternal nutritional status is known to be an important determinant of birthweight, to our knowledge, this study is the first to report associations between the MUAC of Cambodian pregnant women and the risk to stunting in their infants. Stunting during childhood is associated with negative effects, both short‐ and long‐term, on health and development (Black et al., 2013); hence, interventions to improve linear growth and prevent stunting are highly needed. The current findings support the inclusion of pregnant women in programs to reduce stunting prevalence in childhood. Frequently monitoring of MUAC of pregnant women could serve as a simple proxy indicator for identifying infants at risk of growth faltering and stunting. Compared with body mass index, MUAC is a better measurement to determine nutritional status during pregnancy (Dhar & Bhadra, 2008; Elshibly & Schmalisch, 2008; Ogbonna et al., 2007; Sebayang, Dibley, Kelly, Shankar, & Shankar, 2012; Sen, Roy, & Mondal, 2010). Furthermore, MUAC is an easy method to use and does not require sophisticated equipment. A study in Bangladesh (Mridha et al., 2016) showed that providing a lipid‐based supplement to pregnant women, instead of standard iron–folic acid supplements, resulted not only in a higher birthweight (+41 g) but also reduced the prevalence of stunting at birth by almost 4% (from 22.6% to 18.7%). In our study, over 10% of the infants were already stunted within the first months of life. And even though this prevalence of stunting is only half of the stunting prevalence reported by Mridha in their study in Bangladesh, more attention should be given to growth faltering in utero.

The mechanisms by which maternal undernutrition could affect infant linear growth are diverse and include intrauterine undernourishment of foetus, leading to lower birthweight (Imdad & Bhutta, 2012); genetic imprinting, leading to a lower growth potential of the foetus (Martorell & Zongrone, 2012); and reduced nutrient stores given to the infant at birth and during lactation (van Uitert & Steegers‐Theunissen, 2012). Regardless of the relative importance of the underlying mechanisms, improving nutritional status of pregnant women could be an important intervention beside strategies that focus on mothers and their infants between 0 and 6 months of age. A study in Guinea Bissau showed that 3‐month provision of ready‐to‐use supplementary foods to mothers significantly increased MUAC by on average 0.6 cm (Schlossman et al., 2017). The impact of a ready‐to‐use supplementary foods given to pregnant Cambodian women on MUAC needs to be established. In Cambodia, even though exclusive breastfeeding prevalence is relatively good with 60% of infants living in rural areas still being exclusively breastfed at 4 months of age (Som et al., 2018), introduction of water and/or liquid foods before 6 months of age are common and by the age of 6 months, less than 20% of infants remains exclusively breastfed (Som et al., 2018).

In this study, maternal MUAC during the last trimester of pregnancy was associated with the length‐for‐age z‐scores of infants in the multivariate linear regression models but not associated with stunting in the adjusted logistic regression models. These apparent different findings can probably be attributed to the fact that stunting is defined as a cut‐off, and while the majority of infants had a negative LAZ, indicating the presence of undernutrition, most children did not yet reach this cut‐off point classifying them as stunted.

Of concern is the low dietary diversity of pregnant women in Cambodia. As reported earlier, the diet of women of reproductive age is mainly based on rice, vegetables, and a little fish (Som et al., 2018). This dietarian pattern does not change during pregnancy (Som et al., 2018) when energy and nutrient intake should increase to allow for optimal growth of the foetus (Morisaki et al., 2018; Williamson, 2006).

Notwithstanding, there is documented evidence on interventions aimed at improving dietary diversity among women and children in Cambodia. Notable among these interventions is the Enhanced Homestead Food Production programme implemented by the Helen Keller International, a programme which integrates both nutrition specific and nutrition sensitive interventions. The programme targets women and children under 2 years and is aimed at increasing year‐round availability and intake of diverse micronutrient‐rich foods and promotes optimal nutrition and hygiene practices among poor households (Haselow, Stormer, & Pries, 2016). Impact evaluations of the programme revealed its success in improving dietary diversity (Olney, Talukder, Iannotti, Ruel, & Quinn, 2009), household food security, and nutrition status of young children and women in poor populations (Talukder et al., 2010). These findings suggest that deliberate scale‐up of this and other similar programmes, coupled with intensive education and behavioural change communication, can help improve the consumption of more diversified diets among pregnant women.

Our study supports already available evidence that maternal educational status relates with child stunting (Abuya, Ciera, & Kimani‐Murage, 2012; Makoka, 2013; Miller & Rodgers, 2009). A mother who has a higher level of education is more likely to be equipped with child care knowledge and purchasing power to ensure appropriate hygiene and feeding practices. Finally, to better understand the impact of maternal MUAC during pregnancy on stunting, measurement of maternal MUAC at different moments during pregnancy such as during the first, second, and third trimesters should be conducted.

## 5 CONCLUSION

The prevalence of stunting is still unacceptably high in Cambodia, with >30% of children under 5 years of age being affected (National Institute of Statistics, 2015). This study showed that undernutrition of women and foetuses during pregnancy impacts infants linear growth, with >10% of infants already being stunted during the first months of life. Therefore, interventions that aim to tackle stunting should integrate interventions that improve maternal nutritional status (e.g., through the provision of lipid‐based supplements in at‐risk pregnancies) during standard antenatal care of pregnancy or even starting before conception.

## CONFLICTS OF INTEREST

The authors declare no conflict of interest. The opinions and statements in this article are those of the authors and may not reflect official policies or opinions of the organizations they belong to.

## CONTRIBUTIONS

J.B., E.P., A.L., F.T.W. – developed the survey design. S.V.S. – supervised data collection and curated data. D.E.K. – conceived and designed the analysis, performed statistical analysis and prepared the original draft of the manuscript. A.L., S.N.B., and E.P. – supervised the study. All authors read, commented, revised and approved the final manuscript.

## ETHICAL APPROVAL

Ethical approval for the study was obtained from the Cambodia National Ethical Committee for Health Research with the file number 117/NECHR. Informed consent was obtained from all participants, with consent obtained from parents or guardians for participating children.

## Supporting information

Data S1. Table 5: Characteristics of infants aged between 0 to 3.5 months with mean LAZ
