# Supplementary material for: Maternal mid‐upper arm circumference during pregnancy and linear growth among Cambodian infants during the first months of life
Source: Matern Child Nutr. 2020 Aug 24;16(Suppl 2):e12951. doi: 10.1111/mcn.12951 (PMC7591302; doi:10.1111/mcn.12951)
Supplement: Supplementary file 1 — Data S1. Table 5: Characteristics of infants aged between 0 to 3.5 months with mean LAZ [file MCN-16-e12951-s001.docx]

**Supplementary Appendix:**

**Table 5: Characteristics of infants aged between 0 to 3.5 months with mean LAZ**

| **Factors** | **Category** | **Overall*** | **Stunted** | | **Not stunted** | | ***p-value*** |
| --- | --- | --- | --- | --- | --- | --- | --- |
|  |  |  | **n (%)** | **Mean LAZ** | **n (%)** | **Mean LAZ** |  |
| **Sex of child** | Male | 419 (53.8) | 42 (10.0) | -2.40 **±** 0.3 | 377 (90.0) | -0.5 ± 1.0 | 0.096 |
|  | Female | 360 (46.2) | 50 (13.9) | -2.74 **±** 0.6 | 310 (86.1) | -0.4 ± 1.0 |  |
| **Mother’s MUAC at pregnancy** | MUAC < 23.0 cm | 177 (22.7) | 29 (16.4) | -2.77 **±** 0.6 | 148 (83.6) | -0.7 ± 0.8 | **0.032** |
|  | MUAC ≥ 23.0 cm | 602 (77.3) | 63 (10.5) | -2.50 **±** 0.4 | 539 (89.5) | -0.4 ± 1.0 |  |
| **Mother’s education at pregnancy**** | No formal education | 172 (23.0) | 26 (15.1) | -2.76 **±** 0.5 | 146 (84.9) | -0.6 ± 1.0 | 0.059 |
|  | Primary school education | 294 (39.3) | 37 (12.6) | -2.45 **±** 0.3 | 257 (87.4) | -0.5 ± 1.0 |  |
|  | Secondary school and above | 282 (37.7) | 23 (8.2) | -2.59 **±** 0.6 | 259 (91.8) | -0.3 ± 1.1 |  |
| **Received nutrition information at pregnancy?** | Yes | 360 (46.2) | 48 (13.3) | -2.53 ± 0.5 | 312 (86.7) | -0.5 ± 1.1 | 0.222 |
|  | No | 419 (53.8) | 44 (10.5) | -2.64 ± 0.5 | 375 (89.5) | -0.5 ± 0.9 |  |
| **Antenatal care attandance** | Yes | 682 (87.6) | 77 (11.2) | -2.59 ± 0.5 | 605 (88.8) | -0.5 ± 1.0 | 0.233 |
|  | No | 97 (12.4) | 15 (15.5) | -2.55 ± 0.4 | 82 (84.5) | -0.6 ± 1.1 |  |
| **Child being exclusively breastfed?** | Yes | 696 (89.3) | 83 (11.9) | -2.58 ± 0.5 | 613 (88.1) | -0.5 ± 1.0 | 0.773 |
|  | No | 83 (10.7) | 9 (10.8) | -2.60 ± 0.6 | 74 (89.2) | -0.3 ± 1.1 |  |
| **Household wealth index**** | Poorest (1st quintile) | 172 (23.24) | 18 (10.5) | -2.71 ± 0.6 | 154 (89.5) | -0.5 ± 1.1 | 0.247 |
|  | Poorer (2nd quintile) | 165 (22.30) | 26 (15.8) | -2.70 ± 0.5 | 139 (84.2) | -0.5 ± 0.9 |  |
|  | Middle (3rd quintile) | 154 (20.81) | 19 (12.3) | -2.42 ± 0.3 | 135 (87.7) | -0.4 ± 1.0 |  |
|  | Richer (4th quintile) | 151 (20.41) | 15 (9.9) | -2.41 ± 0.3 | 136 (90.1) | -0.4 ± 1.0 |  |
|  | Richest (5th quintile) | 98 (13.24) | 7 (7.1) | -2.66 ± 0.7 | 91 (92.9) | -0.5 ± 0.9 |  |
| **Region** | Phnom Penh | 178 (22.85) | 14 (7.9) | -2.47 ± 0.5 | 164 (92.1) | -0.4 ± 0.9 | 0.063 |
|  | North-East | 601 (77.15) | 78 (13.0) | -2.61 ± 0.5 | 523 (87.0) | -0.5 ± 1.0 |  |

*=Total sample =779; n=number of infants in each category; **=have missing values; *p –values* were derived from chi square test; *p -value* is statistically significant at *p* < 0.05
